# Supplementary material for: Metabolic dependencies of metastasis-initiating cells in female breast cancer
Source: Nat Commun. 2023 Nov 4;14:7076. doi: 10.1038/s41467-023-42748-8 (PMC10625534; doi:10.1038/s41467-023-42748-8)
Supplement: Supplementary file 1 — Supplementary Information [file 41467_2023_42748_MOESM1_ESM.pdf]

# Supplementary Figure 1

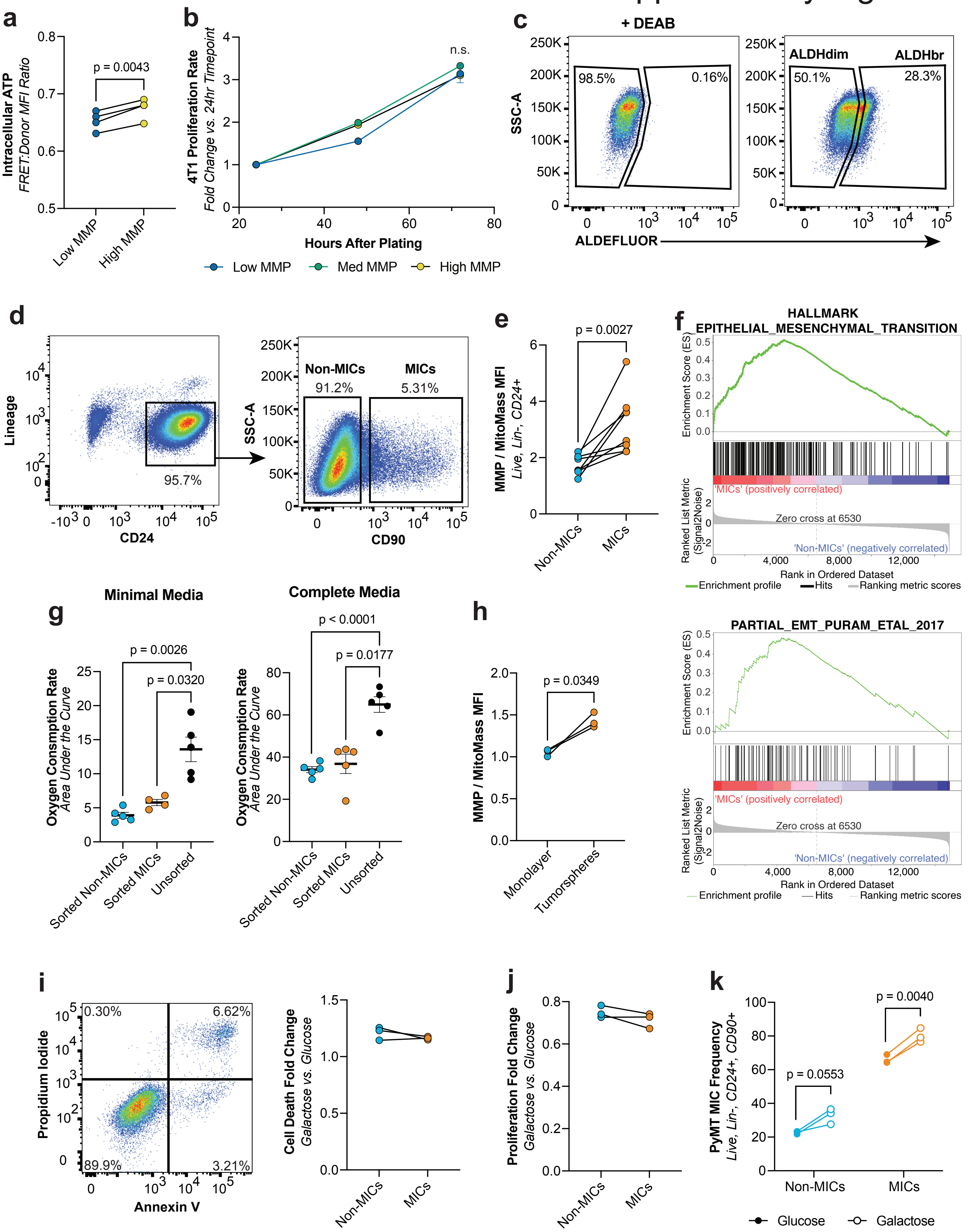

*Supplementary Figure 1: MICs have heightened mitochondrial activity*

a) Intracellular ATP concentrations measured via FRET in high vs. low MMP 4T1 cells. (n=4[a], paired t-test)
b) Proliferation rate of 4T1 cells sorted for low, med, and high MMP normalized to the first measured timepoint. (n=3, ordinary one-way ANOVA)
c) Gating strategy to identify $ALDH^{bright}$ (ALDHbr) vs. $ALDH^{dim}$ (ALDHbr) 4T1 cells. DEAB control is used to determine the boundary between ALDHbr vs. dim.
d) Gating strategy for PyMT MICs and non-MICs. PyMT MICs are identified as CD45-CD31-Ter119-(Lin-), CD24+, and CD90+. PyMT non-MICs are Lin-, CD24+, and CD90-.
e) MMP MFI normalized to mitochondrial mass (MitoMass) MFI in *ex vivo* PyMT non-MICs vs. MICs. (n=8[a], paired t-test)
f) Gene set enrichment plots for EMT and partial EMT in *ex vivo* PyMT MICs vs. non-MICs. (n=3[a])
g) OCR area under the curve (AUC) for sorted vs. unsorted cells, left: in conditions where only glucose is provided as fuel, right: in conditions where complete media is provided (n≥4[a], paired t-test).
h) MMP MFI normalized to MitoMass in monolayer vs. tumorspheres. (n=3[a], paired t-test)
i) Cell death frequency measured by AnnexinV and propidium iodide staining in sorted PyMT non-MICs or MICs cultured for 36 hours in galactose- vs. glucose-containing cell culture media. Left: cells were considered viable when negative for Annexin V and propidium iodide. Right: quantification; expressed as a fold change over the glucose condition. (n=3[a], paired t-test)
j) Proliferation measured by CellTrace Violet staining in sorted PyMT non-MICs or MICs cultured for 36 hours in galactose- vs. glucose-containing cell culture media. Expressed as a fold change over the glucose condition. (n=3[a], paired t-test)
k) MIC frequency in sorted PyMT non-MICs or MICs after 36 hours of culturing in galactose- vs. glucose-containing cell culture media. (n=3[a], paired t-test)

MFI = mean fluorescence intensity. Paired t-test were two-tailed and by ratio. Values shown correspond to means +/- SEM. Source data are provided in the source data file. [a] signifies number of independent experiments or tumors.

# Supplementary Figure 2

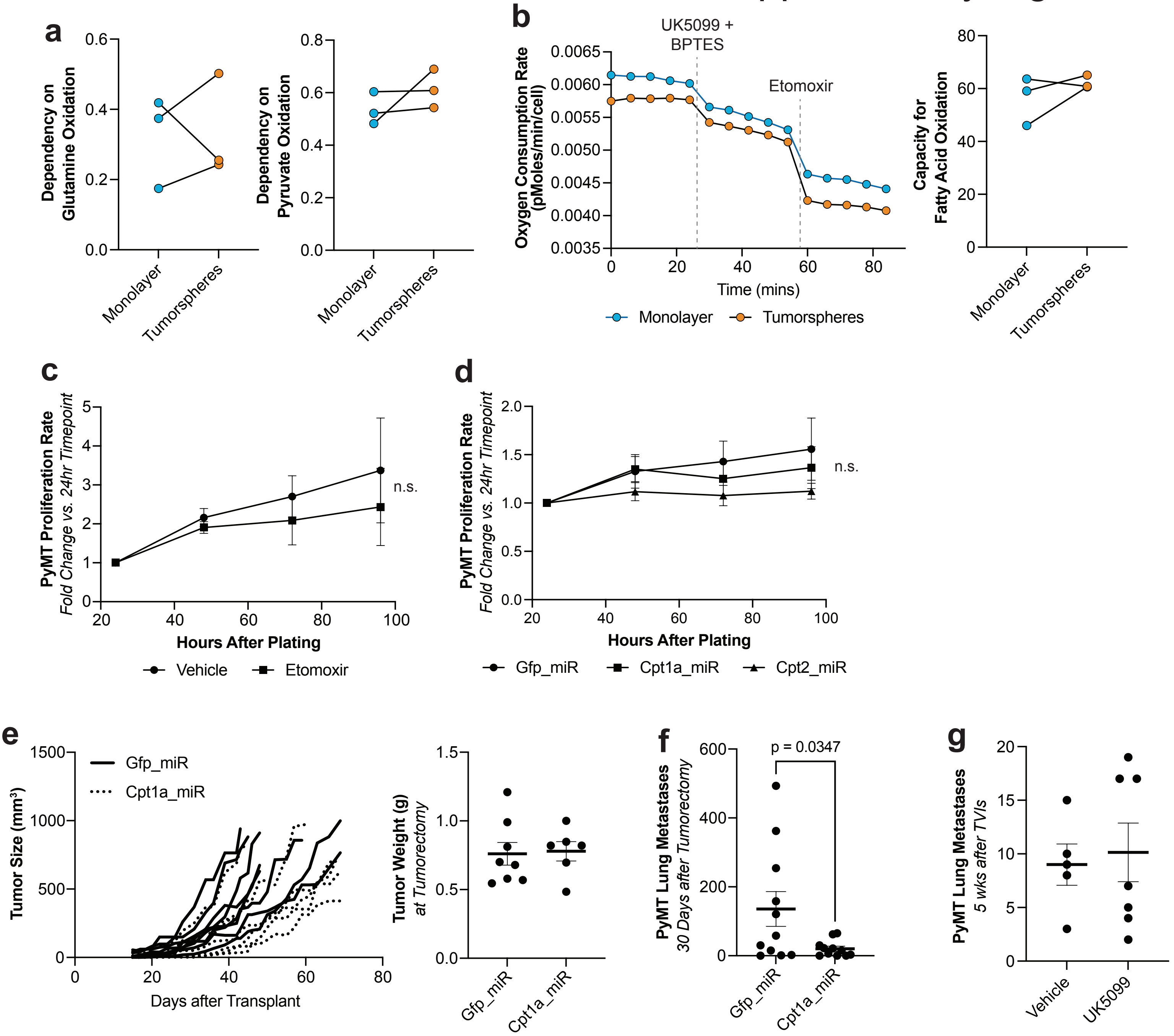

*Supplementary Figure 2: MICs specifically rely on fatty acid usage for metastasis and not for proliferation*

a) Dependence on glutamine (left) and pyruvate (right) oxidation calculated as described in the methods. (n=3[a], paired t-test)
b) Left: OCR over time during a mito fuel flex test in tumorsphere- vs. monolayer-grown cells (normalized to cell number). Right: capacity to undergo LCFA oxidation calculated as described in the methods. (n=3[a], paired t-test)
c) Proliferation rates of PyMT cells after treatment with etomoxir. (n=3[a], unpaired t-test)
d) Proliferation rates of PyMT cells containing KDs of *Gfp*, *Cpt1a*, and *Cpt2*. (n=6[a], unpaired t-test)
e) Left: growth rates of tumors containing miR-mediated KDs of *Gfp* or *Cpt1a*. 72% KD efficiency for *Cpt1a*. Right: tumor weights at time of sacrifice. (n≥7, representative of two independent experiments, unpaired t-test)
f) Resulting spontaneous macrometastases 30 days after removal of tumors containing miR-mediated KDs of *Gfp* or *Cpt1a*. (n=11, combination of two independent experiments, unpaired t-test)
g) Lung metastases in recipient wildtype FVB/N mice after tail vein injection of PyMT cells treated with vehicle or UK5099. (n=5, n=7, respectively, unpaired t-test)

MFI = mean fluorescence intensity. Paired t-test were two-tailed and by ratio. Unpaired t-test were parametric and two-tailed. Values shown correspond to means +/- SEM. Source data are provided in the source data file. [a] signifies number of independent experiments or tumors.

# Supplementary Figure 3

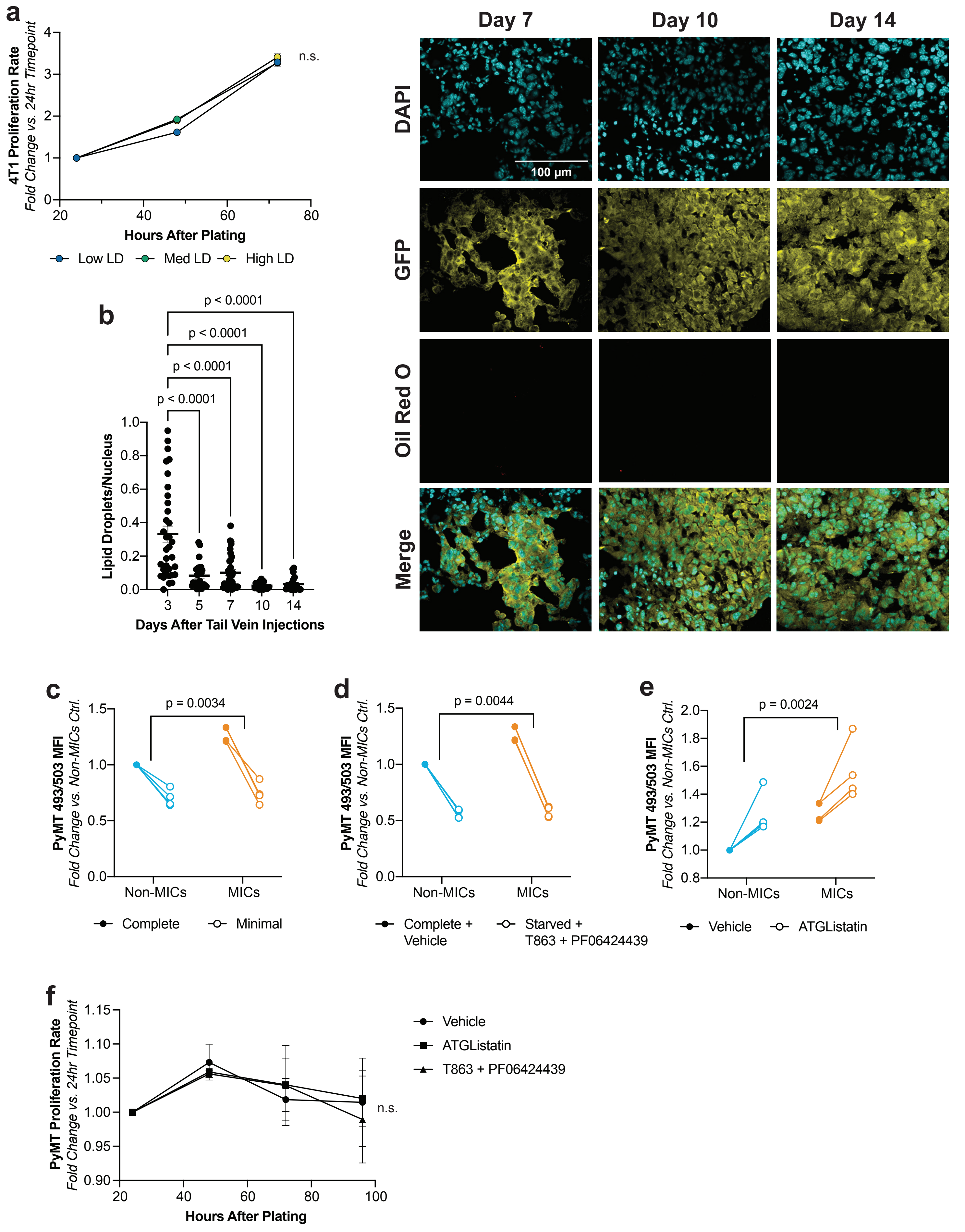

*Supplementary Figure 3: MICs have enhanced lipid droplet dynamics*

a) Proliferation rates of 4T1 cells sorted for low, med, and high BODIPY 493/503 MFI normalized to the first measured timepoint. (n=3, ordinary one-way ANOVA)
b) Number of lipid droplets, measured by oil red O staining, normalized to number of detected nuclei, measured by DAPI staining at specified timepoints after TVI of GFP+ 4T1 cells into recipient DEREG mice. Right: representative images from each timepoint. (n≥18, ordinary one-way ANOVA followed by Dunnett's test)
c) Change in lipid stores in PyMT MICs vs. non-MICs upon starvation for 48 hours. (n=4[a], paired t-test of differences between minimal and complete media conditions)
d) Change in lipid stores in PyMT MICs vs. non-MICs upon starvation and inhibition of lipid droplet generation (using T863 and PF-06424439) for 48 hours. (n=4[a], paired t-test of differences between complete media with vehicle control and minimal media with inhibitor conditions)
e) Change in lipid stores in PyMT MICs vs. non-MICs upon inhibition of lipolysis via treatment with ATGListatin for 48 hours. (n=4[a], paired t-test of differences between complete media with vehicle control and minimal media with inhibitor conditions)
f) Proliferation rates of PyMT cells after pre-treatment with vehicle, ATGListatin, or T863 and PF-06424439. (n=3[a], unpaired t-test)

Paired t-test were two-tailed and by ratio. Unpaired t-test were parametric and two-tailed. Values shown correspond to means +/- SEM. Source data are provided in the source data file.
[a] signifies number of independent experiments or tumors.

# Supplementary Figure 4

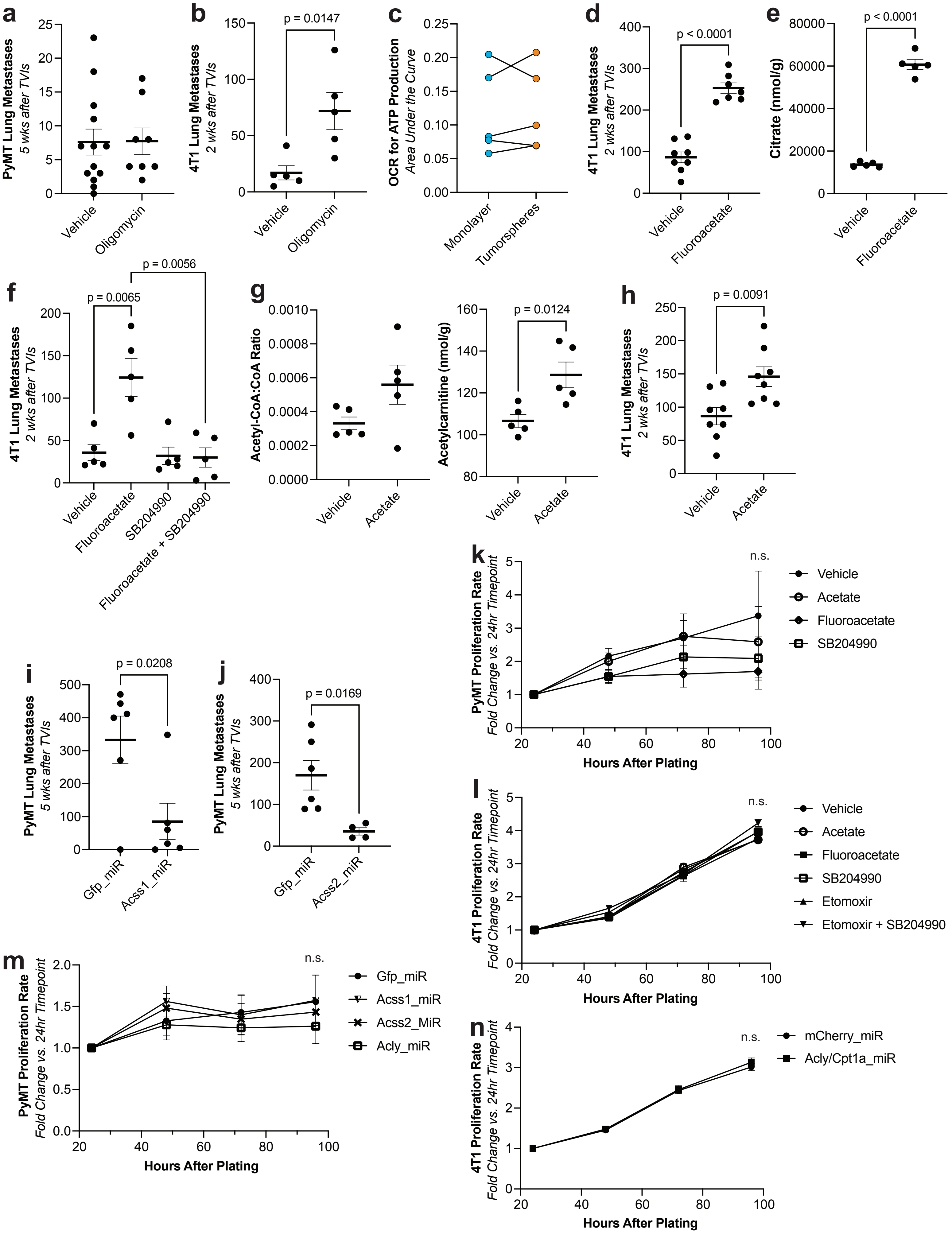

*Supplementary Figure 4: Dependence on acetyl-CoA generation pathways is not due to ATP production or proliferation*

a) Lung metastases in recipient wildtype (wt) FVB/N mice after TVI of PyMT cells treated with oligomycin. (n=13, n=8, respectively, unpaired t-test)
b) Lung metastases in recipient wt Balb/c mice after TVI of 4T1 cells treated with oligomycin. (n=5, unpaired t-test)
c) Area under the OCR curve corresponding to ATP production for monolayer- vs. tumorsphere-grown cells during the mito stress test. (n=5[a], paired t-test)
d) Lung metastases in recipient wt Balb/c mice after TVI of 4T1 cells treated with fluoroacetate. (n=8, n=7, respectively, unpaired t-test)
e) Citrate concentration in 4T1 cells after treatment with fluoroacetate. (n=5, unpaired t-test)
f) Lung metastases in recipient wt Balb/c mice after TVI of 4T1 cells treated with fluoroacetate +/- SB204990. (n=5, unpaired t-test)
g) Acetyl-CoA to CoA ratio (left) and acetylcarnitine concentration (right) in 4T1 cells after acetate treatment. (n=5, unpaired t-test)
h) Lung metastases in recipient wt Balb/c mice after TVI of 4T1 cells treated with acetate. (n=8, unpaired t-test)
i) Lung metastases in recipient wt FVB/N mice after TVI of PyMT cells containing miR-mediated KDs of *Gfp* or *Acss1*. 87% KD efficiency for *Acss1*. (n=6, unpaired t-test)
j) Lung metastases in recipient wt FVB/N mice after TVI of PyMT cells containing miR-mediated KDs of *Gfp* or *Acss2*. 89% KD efficiency for *Acss2*. (n=6, n=4, respectively, unpaired t-test)
k) Proliferation rate of PyMT cells after respective treatments normalized to the first timepoint. (n=3[a], unpaired t-test)
l) Proliferation rate of 4T1 cells after respective treatments normalized to the first timepoint. (n=6, unpaired t-test)
m) Proliferation rate of PyMT cells containing miR-mediated KDs of listed genes normalized to the first timepoint. (n=6[a], unpaired t-test)
n) Proliferation rate of 4T1 cells containing miR-mediated KDs of *mCherry* or *Acly* and *Cpt1a* normalized to the first timepoint. 84% KD efficiency for *Acly*, 71% KD efficiency for *Cpt1a*. (n=6, unpaired t-test)

Paired t-test were two-tailed and by ratio. Unpaired t-test were parametric and two-tailed. Values shown correspond to means +/- SEM. Source data are provided in the source data file.
[a] signifies number of independent experiments or tumors.

# Supplementary Figure 5

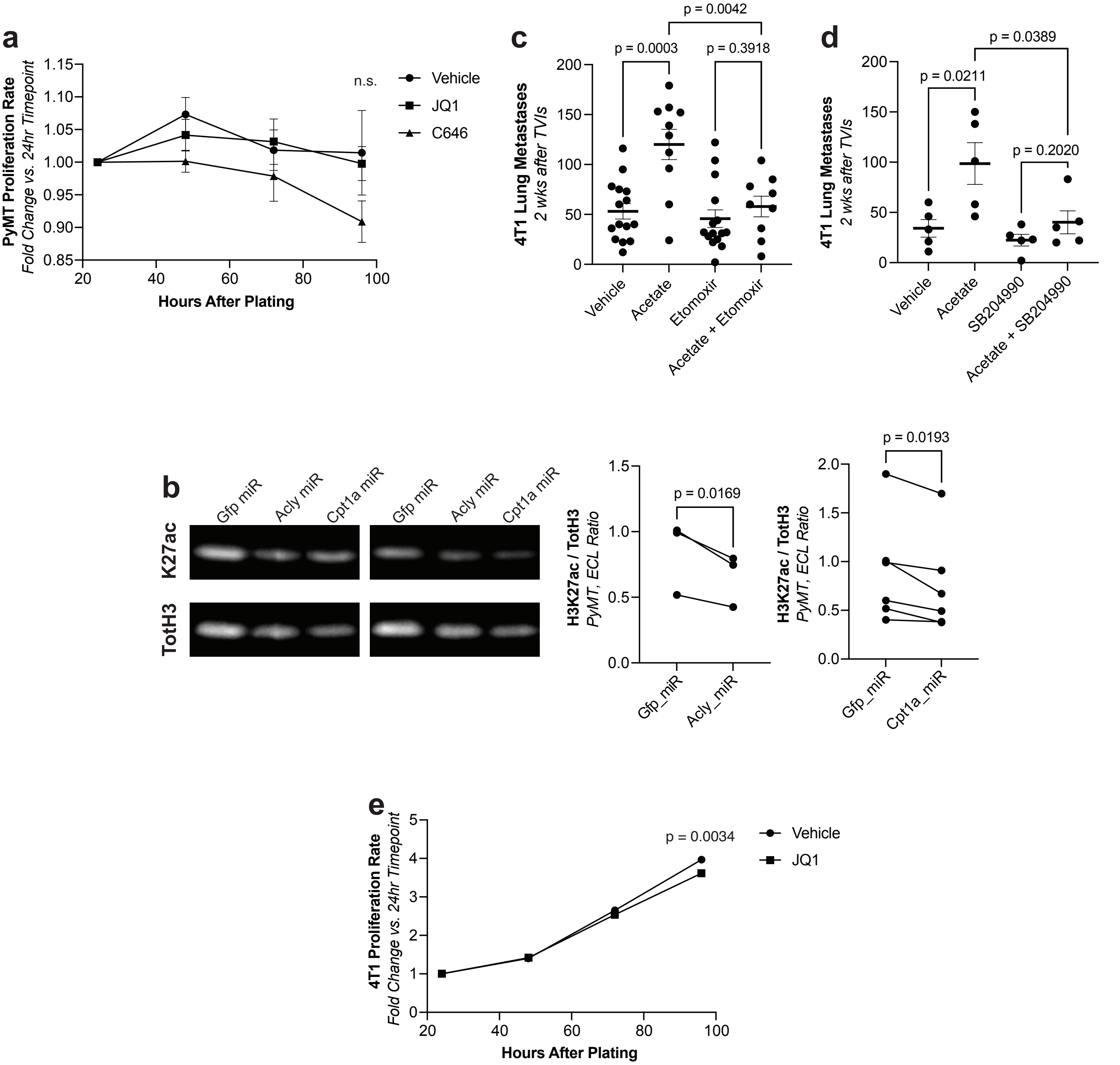

*Supplementary Figure 5: Blocking acetyl-CoA generation and writing and reading of H3K27ac reduces metastasis without affecting proliferative capacity*

a) Proliferation rates of PyMT cells after treatment with vehicle, JQ1, or C646 normalized to the first measured timepoint. (n=3[a], unpaired t-test)
b) Left: Representative Western blots from PyMT cells containing miR-mediated KDs of *Gfp*, *Acly*, or *Cpt1a*. Right: Quantification of the ratio of H3K27ac (K27ac) signal vs. total histone 3 (TotH3) signal in PyMT cells containing miR-mediated KDs of *Acly* (left) or *Cpt1a* (right) vs. *Gfp* KD. (n=3[a], n=6[a], respectively, paired t-test). KD efficiencies are ≥70% and ≥60%, respectively.
c) Lung metastases in recipient wt Balb/c mice after TVI of 4T1 cells after treatment with vehicle or acetate +/- etomoxir. (n≥9, unpaired t-test)
d) Lung metastases in recipient wt Balb/c mice after TVI of 4T1 cells after treatment with vehicle or acetate +/- SB204990. (n=5, unpaired t-test)
e) Proliferation rates of 4T1 cells after treatment with vehicle or JQ1 normalized to the first measured timepoint. (n≥6, unpaired t-test)

Paired t-test were two-tailed and by ratio. Unpaired t-test were parametric and two-tailed. Values shown correspond to means +/- SEM. Source data are provided in the source data file.
[a] signifies number of independent experiments or tumors.

# Supplementary Figure 6

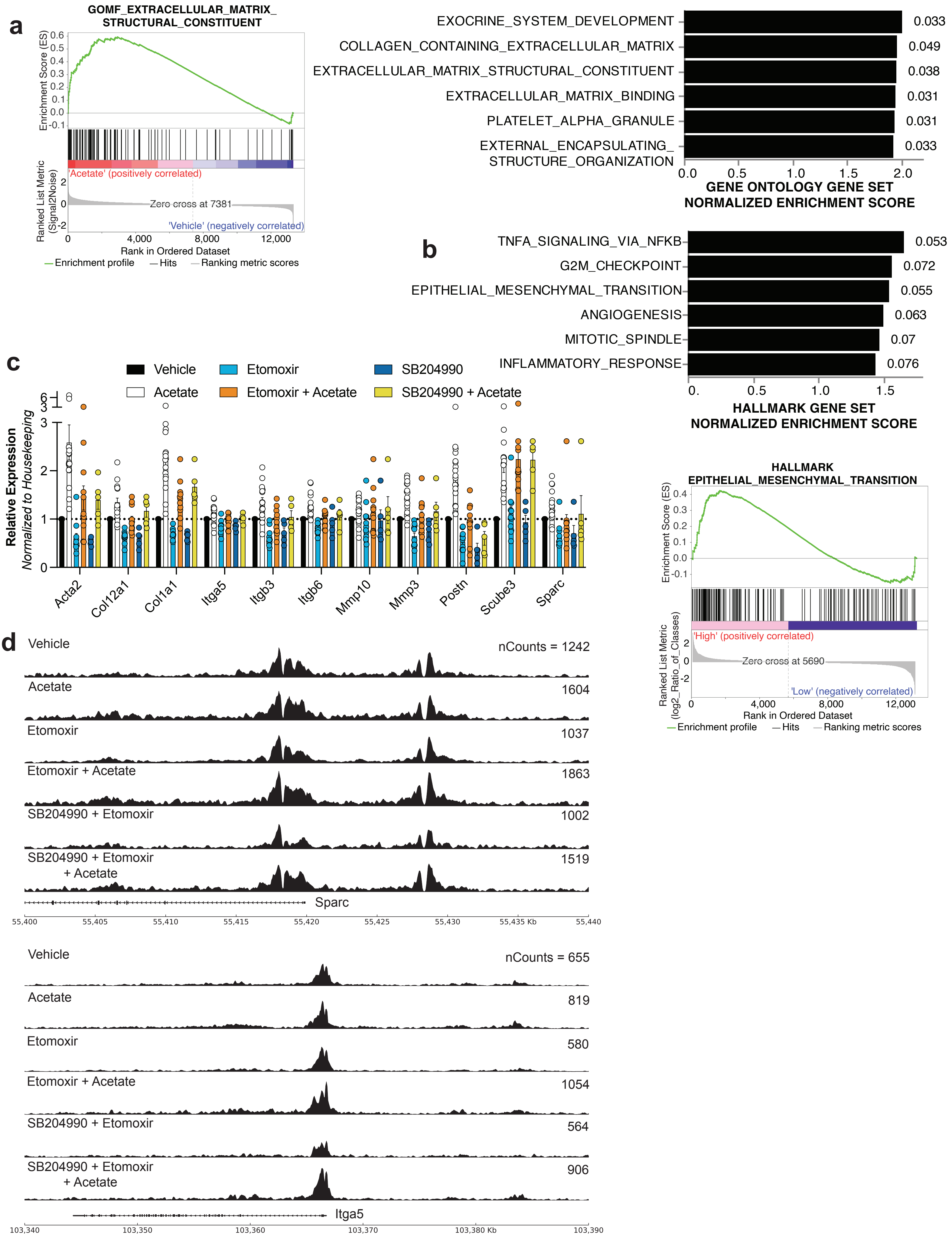

*Supplementary Figure 6: Additional data showing increased EMT gene expression mediated by acetyl-CoA generating pathways and histone acetylation*

a) Left: gene set enrichment plot for the GOMF_EXTRACELLULAR_MATRIX_STRUCTURAL_COMPONENT signature in 5 mM acetate- vs. vehicle-treated 4T1. Right: top upregulated gene sets from the Gene Ontology Collection in the molecular signatures database (mSigDB) in acetate- vs. vehicle-treated 4T1 cells. Numbers beside the bars are the false discovery rates. (n=4[a])
b) Top: top upregulated gene sets from the Hallmark Collection in mSigDB in 4T1 cells sorted for high vs. low MMP. Numbers beside the bars are the false discovery rates. Bottom: gene set enrichment plot for the HALLMARK_EPITHELIAL_MESENCHYMAL_TRANSITION in 4T1 cells sorted for high vs. low MMP. (n=2[a])
c) Relative expression of EMT-related genes in 4T1 cells in response to the indicated treatments normalized to housekeeping genes and plotted as a fold change against vehicle-treated. (n≥5[a]). Values shown correspond to means +/- SEM.
d) H3K27ac ChIP-Seq tracks for *Sparc* (top) and *Itga5* (bottom) from 4T1 cells in response to the indicated treatments. Numbers on the right-hand side of the tracks indicate the associated scaled counts per gene.

Unpaired t-test were parametric and two-tailed. Values shown correspond to means +/- SEM. Source data are provided in the source data file. [a] signifies number of independent experiments or tumors.

# Supplementary Figure 7

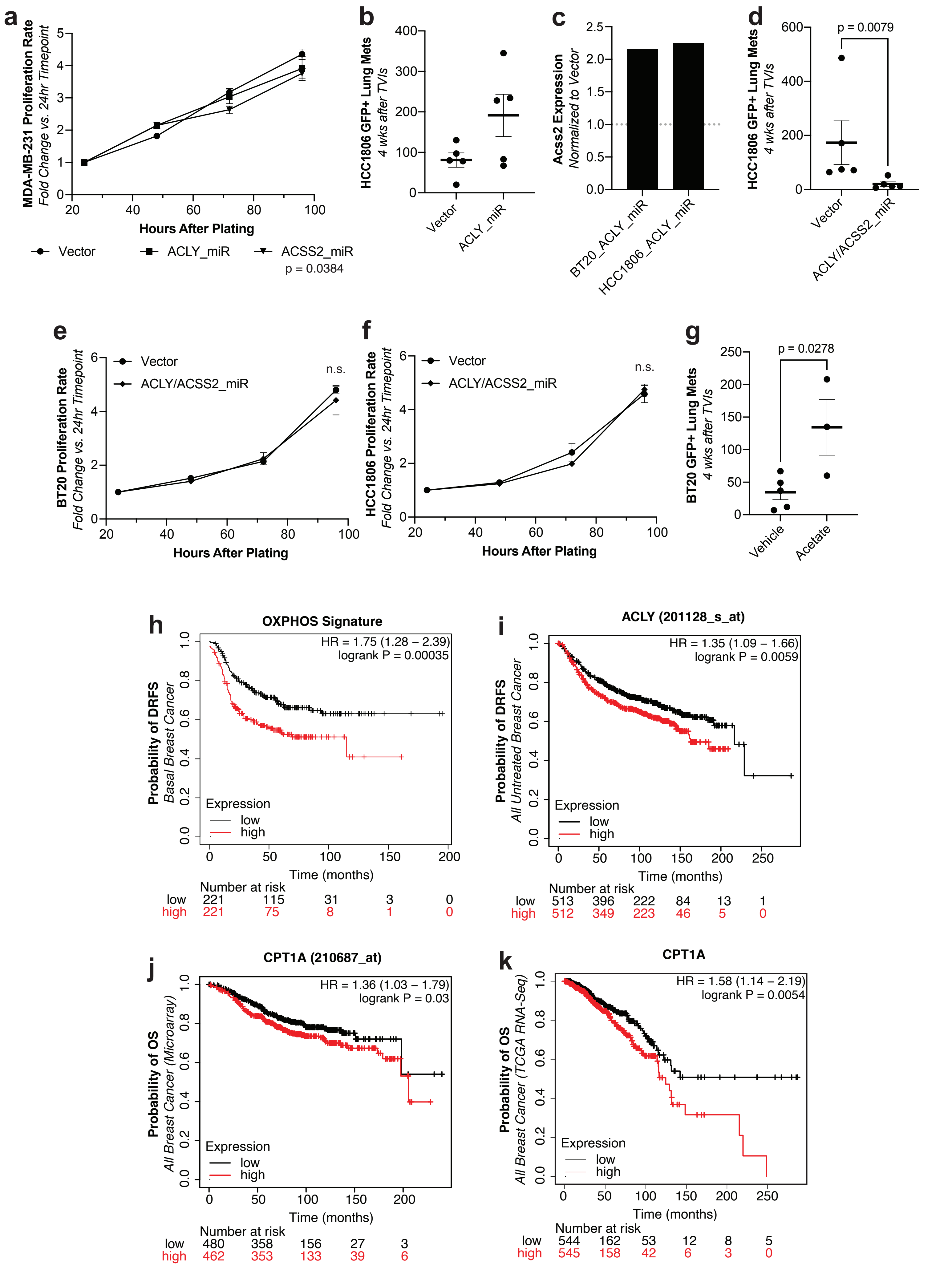

*Supplementary Figure 7: Similar dependence on mitochondrial activity and acetyl-CoA generation in human cell lines and patient cohorts*

a) Proliferation of MDA-MB-231 cells expressing miR-mediated KDs of *ACLY* or *ACSS2*. 80% and 75% KD efficiencies, respectively. Shown as fold change of the first timepoint. (n=4, unpaired t-test)
b) GFP+ metastases in recipient Rag2$^{-/-}\gamma c^{-/-}$ mice after TVI of HCC1806 cells expressing miR-mediated KD of *ACLY*. 83% KD efficiency (n=5, unpaired t-test)
c) Relative expression of *ACSS2* upon miR-mediated KD of *ACLY* in BT20 and HCC1806 cells normalized to vector-only controls. 81% and 83% KD efficiencies, respectively.
d) GFP+ metastases in recipient Rag2$^{-/-}\gamma c^{-/-}$ mice after TVI of HCC1806 cells expressing miR-mediated KDs of *ACLY* and *ACSS2*. 76% and 73% KD efficiencies, respectively. (n=5, unpaired t-test)
e) Proliferation of BT20 cells containing miR-mediated KDs of *ACLY* and *ACSS2*. 78% and 73% KD efficiencies, respectively. Shown as fold change of the first timepoint. (n=6, unpaired t-test)
f) Proliferation of HCC1806 cells containing miR-mediated KDs of *ACLY* and *ACSS2*. 76% and 73% KD efficiencies, respectively. Shown as fold change of the first timepoint. (n=6, unpaired t-test)
g) GFP+ metastases in recipient rag2$^{-/-}\gamma c^{-/-}$ mice after TVI of BT20 cells treated with acetate ($n \geq 3$, unpaired t-test)
h) Kaplan-Meier plots depicting distant relapse-free survival (DRFS) of PAM50-classified basal breast cancer patients derived from the microarray cohort of KM plotter, stratified based on mean expression of OXPHOS-related genes and segregated by median cut-off.
i) Kaplan-Meier plots of DRFS of breast cancer patients that have not undergone systemic treatment derived from the microarray cohort of KM plotter. Patients were stratified by *ACLY* expression and segregated by median cut-off.
j) Kaplan-Meier plots of overall survival (OS) of breast cancer derived from the microarray cohort of KM plotter. Patients were stratified by *CPT1A* expression, and the comparison is made between the upper and lower quartiles.
k) Kaplan-Meier plots of OS of breast cancer patients derived from the TCGA dataset. Patients were stratified by *CPT1A* expression and segregated by median cut-off.

Unpaired t-test were parametric and two-tailed. Values shown correspond to means +/- SEM. Source data are provided in the source data file.

**Supplementary Table 1**

**Pearson scores and p-values when correlating the metastatic potential and histone profiles of 20 TNBC lines from the MetMap project.**

| Histone Marks | P-Value | Pearson Correlation |
|---|---|---|
| H3K4me0 | 0.8448 | 0.0455 |
| H3K4me1 | 0.9921 | -0.0023 |
| H3K4me2 | 0.9452 | 0.016 |
| H3K4ac1 | 0.9837 | 0.0048 |
| H3K9me0K14ac0 | 0.4121 | 0.1889 |
| H3K9me1K14ac0 | 0.9797 | -0.0059 |
| H3K9me2K14ac0 | 0.8837 | 0.034 |
| H3K9me3K14ac0 | 0.1731 | 0.3088 |
| H3K9ac1K14ac0 | 0.2015 | 0.2905 |
| H3K9me0K14ac1 | 0.5277 | 0.146 |
| H3K9me1K14ac1 | 0.9272 | 0.0212 |
| H3K9me2K14ac1 | 0.9791 | -0.0061 |
| H3K9me3K14ac1 | 0.3429 | 0.2178 |
| H3K9ac1K14ac1 | 0.3814 | 0.2014 |
| H3K18ac0K23ac0 | 0.8652 | 0.0394 |
| H3K18ac1K23ac0 | 0.1636 | 0.3155 |
| H3K18ac0K23ac1 | 0.7065 | 0.0874 |
| H3K18ac1K23ac1 | 0.3209 | 0.2277 |
| H3K18ac0K23ub1 | 0.1603 | 0.3178 |
| H3K27me0K36me0 | 0.704 | 0.0881 |
| H3K27me0K36me1 | 0.1818 | 0.303 |
| H3K27me0K36me2 | 0.4944 | 0.1578 |
| H3K27me0K36me3 | 0.21 | 0.2853 |
| H3K27me1K36me0 | 0.8268 | 0.0508 |
| H3K27me1K36me1 | 0.0343 | 0.4636 |
| H3K27me1K36me2 | 0.0607 | 0.4159 |
| H3K27me1K36me3 | 0.1721 | 0.3095 |
| H3K27me2K36me0 | 0.3307 | 0.2232 |
| H3K27me2K36me1 | 0.0452 | 0.4413 |
| H3K27me2K36me2 | 0.6122 | 0.1174 |
| H3K27me3K36me0 | 0.8155 | -0.0542 |
| H3K27me3K36me1 | 0.9607 | 0.0115 |
| H3K27ac1K36me0 | 0.0547 | 0.4251 |
| H3K27ac1K36me1 | 0.049 | 0.4346 |
| H3K27ac1K36me2 | 0.0411 | 0.4491 |
| H3K27ac1K36me3 | 0.0362 | 0.4594 |
| H3.3K27me0K36me0 | 0.8008 | 0.0586 |
| H3K56me0 | 0.0412 | 0.4489 |
| H3K56me1 | 0.5709 | 0.1312 |

| | | |
|---|---|---|
| H3K79me0 | 0.1624 | -0.3163 |
| H3K79me1 | 0.366 | -0.2078 |
| H3K79me2 | 0.2621 | -0.2563 |
