# Supplementary material for: Metabolic dependencies of metastasis-initiating cells in female breast cancer
Source: Nat Commun. 2023 Nov 4;14:7076. doi: 10.1038/s41467-023-42748-8 (PMC10625534; doi:10.1038/s41467-023-42748-8)
Supplement: Supplementary file 4 — Reporting Summary [file 41467_2023_42748_MOESM4_ESM.pdf]

nature portfolio

Corresponding author(s): Joerg Huelsken

Last updated by author(s): Jun 30, 2023

Reporting Summary

Nature Portfolio wishes to improve the reproducibility of the work that we publish. This form provides structure for consistency and transparency in reporting. For further information on Nature Portfolio policies, see our Editorial Policies and the Editorial Policy Checklist.

Statistics

For all statistical analyses, confirm that the following items are present in the figure legend, table legend, main text, or Methods section.

n/a	Confirmed	
☐	☒	The exact sample size (*n*) for each experimental group/condition, given as a discrete number and unit of measurement
☐	☒	A statement on whether measurements were taken from distinct samples or whether the same sample was measured repeatedly
☐	☒	The statistical test(s) used AND whether they are one- or two-sided<br>*Only common tests should be described solely by name; describe more complex techniques in the Methods section.*
☒	☐	A description of all covariates tested
☒	☐	A description of any assumptions or corrections, such as tests of normality and adjustment for multiple comparisons
☐	☒	A full description of the statistical parameters including central tendency (e.g. means) or other basic estimates (e.g. regression coefficient) AND variation (e.g. standard deviation) or associated estimates of uncertainty (e.g. confidence intervals)
☐	☒	For null hypothesis testing, the test statistic (e.g. *F*, *t*, *r*) with confidence intervals, effect sizes, degrees of freedom and *P* value noted<br>*Give P values as exact values whenever suitable.*
☒	☐	For Bayesian analysis, information on the choice of priors and Markov chain Monte Carlo settings
☒	☐	For hierarchical and complex designs, identification of the appropriate level for tests and full reporting of outcomes
☐	☒	Estimates of effect sizes (e.g. Cohen's *d*, Pearson's *r*), indicating how they were calculated

Our web collection on statistics for biologists contains articles on many of the points above.

Software and code

Policy information about availability of computer code

Data collection	Raw LC-MS/MS data was processed using the Agilent Quantitative analysis software (version B.07.00, MassHunter Agilent technologies). Images of GFP+ nodules were taken using SharpCap (v3.2). qPCR data was acquired using the QuantStudio Real-Time PCR Software (v1.3, Thermo Fisher Scientific). Flow cytometry data was collected using the BD FACSDiva software (v7.0, BD). Western blot images were acquired using the Fusion Evolution-Capt Edge Software (Vilber). In vivo bioluminescence images were taken using the Living Image Analysis Software (v4.7, Perkin Elmer). Seahorse data were collected using the Seahorse XF96 Pro Analyzer (Agilent Technologies). Confocal images were taken using NIS-Elements software (Nikon). Absorbance measurements were collected using the TECAN SparkControl software (v1.3, TECAN).
Data analysis	Kaplan Meier plots were generated using a web-based tool: kmplot.com; CCLE data were scaled using the StandardScaler function from scikit learn v1.2.1 and analyzed using the SciPy stats package v1.10.1 for pearsonr correlation then plotted using the mark_point function of Altair v4.2.2, GFP+ lung nodules were analyzed using Fiji v2.9.0, Histology images were analyzed using QuPath v0.4.2 and the pre-trained models from StarDist (2D_versatile_fluo 2D_paper_dsb2018). Gene set enrichment analysis was performed using the GSEA software v4.2.3. RNAseq data was aligned using STAR. ChIPseq data were pre-processed using the nf-core/chipseq v1.2.2 workflow, bigwig files were generated using the BamCoverage tool from deepTools v3.5.1. ChIP tracks were plotted using pyGenomeTracks v3.7 and the heatmap was generated using the computeMatrix function in scaled mode and the plotHeatmap function from deepTools. Flow cytometry data was analyzed using FlowJo v10.7.2. Statistical tests were performed using GraphPad prism 9. More detail is given in the manuscript.

For manuscripts utilizing custom algorithms or software that are central to the research but not yet described in published literature, software must be made available to editors and reviewers. We strongly encourage code deposition in a community repository (e.g. GitHub). See the Nature Portfolio guidelines for submitting code & software for further information.

Data

Policy information about availability of data

All manuscripts must include a data availability statement. This statement should provide the following information, where applicable:

- Accession codes, unique identifiers, or web links for publicly available datasets
- A description of any restrictions on data availability
- For clinical datasets or third party data, please ensure that the statement adheres to our policy

Data from the CCLE were downloaded from the depmap portal: https://depmap.org/portal/download/all/, specifically the files labelled "CCLE_GlobalChromatinProfiling_20181130.csv" and "Supplementary Table 02 basal breast cancer cohort met potential.xlsx". Source data underlying each figure are provided with this paper. High-throughout data can be accessed under the GEO SuperSeries GSE220992 (RNA-seq data: GSE220991, GSE236185, and GSE236222; ChIP-Seq data: GSE236223).

Research involving human participants, their data, or biological material

Policy information about studies with human participants or human data. See also policy information about sex, gender (identity/presentation), and sexual orientation and race, ethnicity and racism.

Reporting on sex and gender	n/a
Reporting on race, ethnicity, or other socially relevant groupings	n/a
Population characteristics	n/a
Recruitment	n/a
Ethics oversight	n/a

Note that full information on the approval of the study protocol must also be provided in the manuscript.

Field-specific reporting

Please select the one below that is the best fit for your research. If you are not sure, read the appropriate sections before making your selection.

☒ Life sciences ☐ Behavioural & social sciences ☐ Ecological, evolutionary & environmental sciences

For a reference copy of the document with all sections, see nature.com/documents/nr-reporting-summary-flat.pdf

Life sciences study design

All studies must disclose on these points even when the disclosure is negative.

Sample size	Sample sizes are indicated in each figure. In vivo sample sizes were determined using power calculations, with B=0.9 and p<0.05.
Data exclusions	No data was excluded
Replication	Replicates are shown in the figures
Randomization	Animals were randomized before assignment to experimental groups
Blinding	Mice were assigned a number prior to assignment to a treatment group. The individual analyzing outcome only knew of the assigned number, but not of the treatment group.

Behavioural & social sciences study design

All studies must disclose on these points even when the disclosure is negative.

Study description	*Briefly describe the study type including whether data are quantitative, qualitative, or mixed-methods (e.g. qualitative cross-sectional, quantitative experimental, mixed-methods case study).*
Research sample	*State the research sample (e.g. Harvard university undergraduates, villagers in rural India) and provide relevant demographic information (e.g. age, sex) and indicate whether the sample is representative. Provide a rationale for the study sample chosen. For studies involving existing datasets, please describe the dataset and source.*

Sampling strategy	*Describe the sampling procedure (e.g. random, snowball, stratified, convenience). Describe the statistical methods that were used to predetermine sample size OR if no sample-size calculation was performed, describe how sample sizes were chosen and provide a rationale for why these sample sizes are sufficient. For qualitative data, please indicate whether data saturation was considered, and what criteria were used to decide that no further sampling was needed.*
Data collection	*Provide details about the data collection procedure, including the instruments or devices used to record the data (e.g. pen and paper, computer, eye tracker, video or audio equipment) whether anyone was present besides the participant(s) and the researcher, and whether the researcher was blind to experimental condition and/or the study hypothesis during data collection.*
Timing	*Indicate the start and stop dates of data collection. If there is a gap between collection periods, state the dates for each sample cohort.*
Data exclusions	*If no data were excluded from the analyses, state so OR if data were excluded, provide the exact number of exclusions and the rationale behind them, indicating whether exclusion criteria were pre-established.*
Non-participation	*State how many participants dropped out/declined participation and the reason(s) given OR provide response rate OR state that no participants dropped out/declined participation.*
Randomization	*If participants were not allocated into experimental groups, state so OR describe how participants were allocated to groups, and if allocation was not random, describe how covariates were controlled.*

Ecological, evolutionary & environmental sciences study design

All studies must disclose on these points even when the disclosure is negative.

Study description	*Briefly describe the study. For quantitative data include treatment factors and interactions, design structure (e.g. factorial, nested, hierarchical), nature and number of experimental units and replicates.*
Research sample	*Describe the research sample (e.g. a group of tagged Passer domesticus, all Stenocereus thurberi within Organ Pipe Cactus National Monument), and provide a rationale for the sample choice. When relevant, describe the organism taxa, source, sex, age range and any manipulations. State what population the sample is meant to represent when applicable. For studies involving existing datasets, describe the data and its source.*
Sampling strategy	*Note the sampling procedure. Describe the statistical methods that were used to predetermine sample size OR if no sample-size calculation was performed, describe how sample sizes were chosen and provide a rationale for why these sample sizes are sufficient.*
Data collection	*Describe the data collection procedure, including who recorded the data and how.*
Timing and spatial scale	*Indicate the start and stop dates of data collection, noting the frequency and periodicity of sampling and providing a rationale for these choices. If there is a gap between collection periods, state the dates for each sample cohort. Specify the spatial scale from which the data are taken*
Data exclusions	*If no data were excluded from the analyses, state so OR if data were excluded, describe the exclusions and the rationale behind them, indicating whether exclusion criteria were pre-established.*
Reproducibility	*Describe the measures taken to verify the reproducibility of experimental findings. For each experiment, note whether any attempts to repeat the experiment failed OR state that all attempts to repeat the experiment were successful.*
Randomization	*Describe how samples/organisms/participants were allocated into groups. If allocation was not random, describe how covariates were controlled. If this is not relevant to your study, explain why.*
Blinding	*Describe the extent of blinding used during data acquisition and analysis. If blinding was not possible, describe why OR explain why blinding was not relevant to your study.*

Did the study involve field work? ☐ Yes ☐ No

Field work, collection and transport

Field conditions	*Describe the study conditions for field work, providing relevant parameters (e.g. temperature, rainfall).*
Location	*State the location of the sampling or experiment, providing relevant parameters (e.g. latitude and longitude, elevation, water depth).*
Access & import/export	*Describe the efforts you have made to access habitats and to collect and import/export your samples in a responsible manner and in compliance with local, national and international laws, noting any permits that were obtained (give the name of the issuing authority, the date of issue, and any identifying information).*
Disturbance	*Describe any disturbance caused by the study and how it was minimized.*

Reporting for specific materials, systems and methods

We require information from authors about some types of materials, experimental systems and methods used in many studies. Here, indicate whether each material, system or method listed is relevant to your study. If you are not sure if a list item applies to your research, read the appropriate section before selecting a response.

Materials & experimental systems

n/a	Involved in the study
☐	☒ Antibodies
☐	☒ Eukaryotic cell lines
☒	☐ Palaeontology and archaeology
☐	☒ Animals and other organisms
☒	☐ Clinical data
☒	☐ Dual use research of concern
☒	☐ Plants

Methods

n/a	Involved in the study
☐	☒ ChIP-seq
☐	☒ Flow cytometry
☒	☐ MRI-based neuroimaging

Antibodies

Antibodies used

Flow cytometry antibodies: Ter119 (Biolegend #116203, clone Ter119, 1:300), CD31 (Biolegend #102503, clone Mec13.3, 1:300), CD45 (Biolegend #103104, clone 30-F11, 1:300), CD24 (Biolegend #101807, clone MI/69, 1:300), CD90.1 (Biolegend #202526, clone 1:300), H3K27ac (CST #8173, clone D5E4, 1:100), Total H3 (Novus Biologicals #NBP2-59277, clone 1B1B2, 1:100), Alexa 488 anti-rabbit secondary (ThermoFisher Scientific #A-21206), Alexa 488 anti-mouse secondary (ThermoFisher Scientific #A-11029). Western blotting antibodies: H3K27ac (CST #8173, clone D5E4, 1:1000), Total H3 (CST #3638, clone 96C10, 1:2000), HRP anti-rabbit secondary (Jackson Immunosearch #711-035-152, 1:10000), HRP anti-mouse secondary (Anti-Mouse: Promega #W4021, 1:10000). ChIP-seq antibody: H3K27ac (CST #8173, clone D5E4, 1:100)

Validation

Validations have been performed by the manufacturer, and can be seen on the manufacturer's website or in the provided technical sheets. Their validation statements are as follows: Biolegend: Specificity testing of 1-3 target cell types with either single- or multi-color analysis (including positive and negative cell types). Once specificity is confirmed, each new lot must perform with similar intensity to the in-date reference lot. Brightness (MFI) is evaluated from both positive and negative populations. Each lot product is validated by QC testing with a series of titration dilutions. Cell Signaling Technologies: All ChIP-seq validated antibodies are first subjected to the ChIP-qPCR validation protocol. Antibody sensitivity for ChIP-seq is then confirmed by analyzing the signal:noise ratio of target enrichment across the genome in antibody:input control comparisons. The antibody must provide an acceptable minimum number of defined enrichment peaks and a minimum signal:noise threshold compared to input chromatin. For sequence-specific DNA-binding transcription factors, antibody specificity is determined by performing motif analysis of enriched chromatin fragments. Antibody specificity is further determined by comparing enrichment across the genome using multiple antibodies against distinct target protein epitopes. Antibody specificity is confirmed using antibodies against different subunits of a multiprotein complex. Antibody specificity is further confirmed by comparing enrichment across the genome to published ChIP-seq data (ie, ENCODE) using additional antibodies for a given target protein. On our side, all flow cytometry experiments are done with an FMO control or secondary only antibody to ensure specific staining and ChIP antibodies were validated against known genes that are regulated by H3K27ac via ChIP-qPCR.

Eukaryotic cell lines

Policy information about cell lines and Sex and Gender in Research

Cell line source(s)

The murine cancer cell line 4T1 was obtained from ATCC, the human cancer cell lines MDA-MB-231 and BT549 were a kind gift from Prof. Paloma Ordonez Moran (University of Nottingham), and the human cancer cell lines, BT20 and HCC1806, were a kind gift from Prof. Cathrin Brisken (EPFL).

Authentication

the cell lines were not authenticated by us

Mycoplasma contamination

tested regularly as negative

Commonly misidentified lines
(See ICLAC register)

No commonly misidentified lines were used in this manuscript

Palaeontology and Archaeology

Specimen provenance

Provide provenance information for specimens and describe permits that were obtained for the work (including the name of the issuing authority, the date of issue, and any identifying information). Permits should encompass collection and, where applicable, export.

Specimen deposition

Indicate where the specimens have been deposited to permit free access by other researchers.

Dating methods | *If new dates are provided, describe how they were obtained (e.g. collection, storage, sample pretreatment and measurement), where they were obtained (i.e. lab name), the calibration program and the protocol for quality assurance OR state that no new dates are provided.*

☐ Tick this box to confirm that the raw and calibrated dates are available in the paper or in Supplementary Information.

Ethics oversight | *Identify the organization(s) that approved or provided guidance on the study protocol, OR state that no ethical approval or guidance was required and explain why not.*

Note that full information on the approval of the study protocol must also be provided in the manuscript.

Animals and other research organisms

Policy information about studies involving animals; ARRIVE guidelines recommended for reporting animal research, and Sex and Gender in Research

Laboratory animals	MMTV-PyMT mice in the FVB/N background and the rag2-/-γc-/- mice in the NMRI background were obtained from JAX and were described previously. DEREG mice in the BALB/c background were used as GFP-tolerant recipients and were obtained by backcrossing DEREG mice in the C57BL/6J background to wildtype BALB/c mice for at least 10 generations. Wildtype FVB mice were bred inhouse, and wildtype BALB/c mice were purchased from Charles River Laboratories. MMTV-PyMT mice used within 12-18 weeks of age. Recipient mice were used between 8-20 weeks of age.<br>All mouse strains were housed in pathogen-free facilities in Lausanne, Switzerland (AIVC at the Agora Cancer Research Center and CPG at EPFL). Mice were placed in IVC cages, 5 mice to a cage, with enrichment, and ad libitum food and water. The facility was on a 12hr dark/light cycle with controlled temperature (22 +/- 2 degrees Celsius) and humidity (55% +/- 10%).
Wild animals	No wild animals were used in this study
Reporting on sex	Since this is a breast cancer study, the experiments were performed in female mice
Field-collected samples	No field collected samples were used in this study
Ethics oversight	All mice were handled according the to Swiss guidelines for animal safety. All animal experiments were done in accordance to the licenses approved and issued by Service de la Consommation et des Affaires Vétérinaires, Canton de Vaud, Switzerland.

Note that full information on the approval of the study protocol must also be provided in the manuscript.

Clinical data

Policy information about clinical studies

All manuscripts should comply with the ICMJE guidelines for publication of clinical research and a completed CONSORT checklist must be included with all submissions.

Clinical trial registration	*Provide the trial registration number from ClinicalTrials.gov or an equivalent agency.*
Study protocol	*Note where the full trial protocol can be accessed OR if not available, explain why.*
Data collection	*Describe the settings and locales of data collection, noting the time periods of recruitment and data collection.*
Outcomes	*Describe how you pre-defined primary and secondary outcome measures and how you assessed these measures.*

Dual use research of concern

Policy information about dual use research of concern

Hazards

Could the accidental, deliberate or reckless misuse of agents or technologies generated in the work, or the application of information presented in the manuscript, pose a threat to:

No	Yes	
☒	☐	Public health
☒	☐	National security
☒	☐	Crops and/or livestock
☒	☐	Ecosystems
☒	☐	Any other significant area

Experiments of concern

Does the work involve any of these experiments of concern:

No	Yes	
☒	☐	Demonstrate how to render a vaccine ineffective
☒	☐	Confer resistance to therapeutically useful antibiotics or antiviral agents
☒	☐	Enhance the virulence of a pathogen or render a nonpathogen virulent
☒	☐	Increase transmissibility of a pathogen
☒	☐	Alter the host range of a pathogen
☒	☐	Enable evasion of diagnostic/detection modalities
☒	☐	Enable the weaponization of a biological agent or toxin
☒	☐	Any other potentially harmful combination of experiments and agents

Plants

Seed stocks	*Report on the source of all seed stocks or other plant material used. If applicable, state the seed stock centre and catalogue number. If plant specimens were collected from the field, describe the collection location, date and sampling procedures.*
Novel plant genotypes	*Describe the methods by which all novel plant genotypes were produced. This includes those generated by transgenic approaches, gene editing, chemical/radiation-based mutagenesis and hybridization. For transgenic lines, describe the transformation method, the number of independent lines analyzed and the generation upon which experiments were performed. For gene-edited lines, describe the editor used, the endogenous sequence targeted for editing, the targeting guide RNA sequence (if applicable) and how the editor was applied.*
Authentication	*Describe any authentication procedures for each seed stock used or novel genotype generated. Describe any experiments used to assess the effect of a mutation and, where applicable, how potential secondary effects (e.g. second site T-DNA insertions, mosiacism, off-target gene editing) were examined.*

ChIP-seq

Data deposition

☒ Confirm that both raw and final processed data have been deposited in a public database such as GEO.

☒ Confirm that you have deposited or provided access to graph files (e.g. BED files) for the called peaks.

Data access links<br>*May remain private before publication.*	GSE236223
Files in database submission	All normalized counts, bigwig files, and raw sequencing files have been deposited for 2 sets of samples with 6 treatment conditions each.
Genome browser session<br>(e.g. UCSC)	https://genome-euro.ucsc.edu/s/cmyoung/itga5, https://genome-euro.ucsc.edu/s/cmyoung/col1a1, https://genome-euro.ucsc.edu/s/cmyoung/sparc, https://genome-euro.ucsc.edu/s/cmyoung/postn

Methodology

Replicates	Two replicates per treatment condition with a minimum of 75% correlation between samples of the same treatment.
Sequencing depth	Samples were sequenced at a minimum sequencing depth of 26.7 M reads per sample on a Novaseq on paired-end mode (PE60) with over 85% unique reads
Antibodies	Acetyl-Histone H3 (Lys27) (D5E4) XP Rabbit mAb #8173 from Cell Signaling Technologies
Peak calling parameters	ChIPseq sampes were pre-processed using nf-core/chipseq v1.2.2 against a merged human and mouse genome. Peak calling with MACS2 was a broad cutoff set to 0.1 at an FDR of 0.05.
Data quality	QC was performed within the nf-core workflow using the multiQC module with FRIP scores between 10-40% based on default MACS2 parameters.
Software	ChIPseq sampes were pre-processed using nf-core/chipseq v1.2.2

Flow Cytometry

Plots

Confirm that:

☒ The axis labels state the marker and fluorochrome used (e.g. CD4-FITC).

☒ The axis scales are clearly visible. Include numbers along axes only for bottom left plot of group (a 'group' is an analysis of identical markers).

☒ All plots are contour plots with outliers or pseudocolor plots.

☒ A numerical value for number of cells or percentage (with statistics) is provided.

Methodology

Sample preparation	Cells were obtained by dissociating fresh mouse tissue or trypsinization from cell culture dishes. Details are described in the paper.
Instrument	Fortessa 2 and Symphony flow cytometers from BD.
Software	Flowjo v10.7.2
Cell population abundance	ex vivo PyMT MICs typically are in the 2-10% range, but this increases over time in the culture and depending on the treatment conditions. For experiments with the 4T1, 5% of each population was always taken. More details for each experiment are outlined in the manuscript.
Gating strategy	Gating strategies for each experiment are outlined in the manuscript and in the supplementary figures.

☒ Tick this box to confirm that a figure exemplifying the gating strategy is provided in the Supplementary Information.

Magnetic resonance imaging

Experimental design

Design type	*Indicate task or resting state; event-related or block design.*
Design specifications	*Specify the number of blocks, trials or experimental units per session and/or subject, and specify the length of each trial or block (if trials are blocked) and interval between trials.*
Behavioral performance measures	*State number and/or type of variables recorded (e.g. correct button press, response time) and what statistics were used to establish that the subjects were performing the task as expected (e.g. mean, range, and/or standard deviation across subjects).*

Acquisition

Imaging type(s)	*Specify: functional, structural, diffusion, perfusion.*
Field strength	*Specify in Tesla*
Sequence & imaging parameters	*Specify the pulse sequence type (gradient echo, spin echo, etc.), imaging type (EPI, spiral, etc.), field of view, matrix size, slice thickness, orientation and TE/TR/flip angle.*
Area of acquisition	*State whether a whole brain scan was used OR define the area of acquisition, describing how the region was determined.*

Diffusion MRI ☐ Used ☐ Not used

Preprocessing

Preprocessing software	*Provide detail on software version and revision number and on specific parameters (model/functions, brain extraction, segmentation, smoothing kernel size, etc.).*
Normalization	*If data were normalized/standardized, describe the approach(es): specify linear or non-linear and define image types used for transformation OR indicate that data were not normalized and explain rationale for lack of normalization.*
Normalization template	*Describe the template used for normalization/transformation, specifying subject space or group standardized space (e.g. original Talairach, MNI305, ICBM152) OR indicate that the data were not normalized.*
Noise and artifact removal	*Describe your procedure(s) for artifact and structured noise removal, specifying motion parameters, tissue signals and physiological signals (heart rate, respiration).*

Volume censoring | *Define your software and/or method and criteria for volume censoring, and state the extent of such censoring.*

Statistical modeling & inference

Model type and settings | *Specify type (mass univariate, multivariate, RSA, predictive, etc.) and describe essential details of the model at the first and second levels (e.g. fixed, random or mixed effects; drift or auto-correlation).*

Effect(s) tested | *Define precise effect in terms of the task or stimulus conditions instead of psychological concepts and indicate whether ANOVA or factorial designs were used.*

Specify type of analysis: ☐ Whole brain ☐ ROI-based ☐ Both

Statistic type for inference | *Specify voxel-wise or cluster-wise and report all relevant parameters for cluster-wise methods.*

(See Eklund et al. 2016)

Correction | *Describe the type of correction and how it is obtained for multiple comparisons (e.g. FWE, FDR, permutation or Monte Carlo).*

Models & analysis

n/a	Involved in the study
☐	☐ Functional and/or effective connectivity
☐	☐ Graph analysis
☐	☐ Multivariate modeling or predictive analysis

Functional and/or effective connectivity | *Report the measures of dependence used and the model details (e.g. Pearson correlation, partial correlation, mutual information).*

Graph analysis | *Report the dependent variable and connectivity measure, specifying weighted graph or binarized graph, subject- or group-level, and the global and/or node summaries used (e.g. clustering coefficient, efficiency, etc.).*

Multivariate modeling and predictive analysis | *Specify independent variables, features extraction and dimension reduction, model, training and evaluation metrics.*
